# Post-ASPECTS based on hyperdensity in NCCT immediately after thrombectomy is an ultra-early predictor of hemorrhagic transformation and prognosis

**DOI:** 10.3389/fneur.2022.887277

**Published:** 2022-08-10

**Authors:** Lulu Chen, Ziqi Xu, Chen Zhang, Yachen Ji, Xianjun Huang, Weimin Yang, Zhiming Zhou, Shuiping Wang, Kai Wang, Benyan Luo, Jingye Wang

**Affiliations:** ^1^Department of Neurology, First Affiliated Hospital of Anhui Medical University, Hefei, China; ^2^Department of Neurology, Brain Medical Centre, First Affiliated Hospital, Zhejiang University School of Medicine, Hangzhou, China; ^3^Department of Neurology, Yijishan Hospital of Wannan Medical College, Wuhu, China

**Keywords:** brain parenchymal hyperdensity, hemorrhagic transformation, Post-ASPECTS, thrombectomy, 90-day prognosis

## Abstract

**Background and Purpose:**

Almost half of the patients exhibit futile recanalization after thrombectomy; however, the early postoperative predictors of futile recanalization remain unclear. We analyzed the relationship of post-thrombectomy ASPECTS (Post-ASPECTS) with 90-day prognosis and hemorrhagic transformation (HT).

**Methods:**

We collected data from patients with acute ischemic stroke (AIS) with anterior-circulation large vessel occlusion (ACLVO) who were treated *via* thrombectomy within 10 h in 3 hospitals. Successful endovascular recanalization was achieved (modified thrombolysis in cerebral ischemia [mTICI] 2b/3). Non-contrast computed tomography (NCCT) examination was performed immediately (within 1 h) after thrombectomy. Post-ASPECTS were scored based on the brain parenchymal hyperdensity in NCCT according to the ASPECTS scoring method. HT was defined according to the ECASS II classification criteria. Linear correlation, logistic regression, and receiver operating characteristic curve analyses were used to determine the influencing factors and best predictive value of 90-day prognosis, 90-day death, and HT.

**Results:**

A total of 231 patients were enrolled. The good prognosis rate, mortality rate, and HT rate were 57.1, 9.5, and 38.3%, respectively. The Post-ASPECTS affected poor prognosis, death, and HT. The best predictive value of Post-ASPECTS for poor prognosis, death, and HT was 7. The specificities of Post-ASPECTS for predicting HT, poor prognosis, and death were 87.6% (AUC, 0.811; *P* < 0.001), 87.1% (AUC, 0.768; *P* < 0.001), and 73.7% (AUC, 0.748; *P* < 0.001), with positive predictive values of 74.2, 75.7, and 21.4%, respectively.

**Conclusion:**

Post-ASPECTS predicted 90-day prognosis, death, and HT with high specificity and high positive predictive value in patients with AIS with ACLVO. Post-ASPECTS may be an ultra-early predictor of prognosis after thrombectomy.

## Introduction

Mechanical thrombectomy is the most effective therapy for patients with acute ischemic stroke (AIS) with anterior-circulation large vessel occlusion (ACLVO) within 24 h of onset (1–3). Complete recanalization is associated with a good prognosis but is not equal to successful reperfusion (4–6). It has been reported that the recanalization rate can reach 58.7–95.0% in clinical trials (7–9). Although many patients (46%) have a good prognosis, others exhibit futile recanalization (10, 11).

Early prediction of prognosis after thrombectomy helps to guide treatment decisions such as suitability for rehabilitation. Therefore, it is imperative to explore the postoperative predictors for futile recanalization, which are valuable for treatment strategies. Rapid neurological improvement (RNI) based on NIHSS score, the state of reperfusion, and infarct volumes helps predict prognosis (4, 12–14). However, some patients cannot undergo NIHSS assessment and reperfusion examination in a short time after thrombectomy for various reasons. In addition, assessing infarct volumes after thrombectomy usually takes several days. Therefore, identifying simple and early post-thrombectomy predictors has important clinical value.

The brain parenchymal hyperdensity shown on non-contrast computed tomography (NCCT) is a common clinical phenomenon after thrombectomy. Previous studies have shown that parenchymal hyperdensity is related to the volume of cerebral infarction and hemorrhagic transformation (HT), but whether it is related to prognosis remains controversial (15–17). The purpose of this study was to analyze the usefulness of post-thrombectomy ASPECTS (Post-ASPECTS) based on brain parenchymal hyperdensity on NCCT within 1 h after thrombectomy in predicting the 90-day prognosis and HT in patients with AIS with ACLVO.

## Materials and methods

This study was approved by the Institutional Review Board at the First Affiliated Hospital of Anhui Medical University. Patient informed consent was waived by our review board given the multicenter retrospective analysis of the acquired anonymous data.

### Study subjects

This retrospective study collected data from patients with AIS with ACLVO treated by thrombectomy within 10 h from the following 3 hospitals: the First Affiliated Hospital of Anhui Medical University (July 2018 to August 2021), the First Affiliated Hospital of Zhejiang University (July 2018 to May 2021), and Yijishan Hospital of Wannan Medical College (August 2018 to December 2020). The inclusion criteria were as follows: (1) age ≥ 18 years; (2) pre-stroke mRS score of 0 to 1; (3) causative occlusion of the internal carotid artery (ICA) or M1 segment of the middle cerebral artery (M1); (4) baseline NIHSS score ≥ 6; (5) pre-thrombectomy ASPECTS (Pre-ASPECTS) ≥ 6; (6) patients whose time from onset to groin puncture (OTP) > 6 h had CTP and complied with the thrombectomy criteria outlined in the guidelines (1); (7) successful endovascular recanalization was achieved (modified thrombolysis in cerebral ischemia [mTICI] 2b/3); (8) postoperative NCCT examination of each patient performed immediately (within 1 h) to obtain Post-ASPECTS; and (9) patients whose basic information, imaging data, and 90-day follow-up data were obtained. The exclusion criteria were as follows: (1) death due to a tumor or other definite causes within 90 days after thrombectomy. (2) bilateral occlusion. (3) artery rupture; or (4) obvious space-occupying effect of brain parenchymal hyperdensity, including the mass effect on the ventricular system, midline shift, and sulcal effacement (such as HT-associated subarachnoid hemorrhage).

### Data collection and analysis

The following data were collected: age, sex, smoking, hypertension, diabetes, atrial fibrillation, baseline NIHSS score, Pre-ASPECTS, intravenous thrombolysis, Post-ASPECTS, OTP time, onset to recanalization (OTR) time, occlusion artery, HT, length of hospital stay, and 90-day mRS score. Brain parenchymal hyperdensity was defined as a higher density increased by at least five Hounsfield units with no significant space-occupying effect compared to that of the unaffected contralateral side on NCCT after thrombectomy within 1 h (18, 19). Post-ASPECTS were scored based on the brain parenchymal hyperdensity in NCCT immediately (within 1 h) after thrombectomy according to the 10 areas and scoring criteria defined in the ASPECTS scoring method (20). The Post-ASPECTS scores were performed independently by two neurologists and, in case of disagreement, agreed upon after discussion with a third neurovascular specialist. HT was detected by NCCT after post-thrombectomy (24 h to 7 days). According to the ECASS II classification criteria, HT was divided into HI1, HI2, PH1, and PH2 (21). The patients were divided into the following subgroups according to different criteria: the 90-day mRS good prognosis group (mRS ≤ 2) and the poor prognosis group (mRS > 2), as well as the 90-day death group, non-death group, HT group, and non-HT group.

### Statistical analysis

The SPSS 23.0 software was used for statistical analysis. Continuous variables are expressed as the median and interquartile distance. Owing to the skewed distribution of the data, comparisons between groups were performed *via* the Mann–Whitney U test. The ordered categorical variables are expressed as the median and interquartile distance, and these groups were compared *via* Mann–Whitney U tests. The unordered categorical variables were expressed as numbers of cases and percentages and were compared *via* chi-square tests. Spearman's correlation analysis was used for correlation analysis. Baseline NIHSS, Pre-ASPECTS, and Post-ASPECTS were included in the logistic regression model separately. Then, other variables with *P* < 0.05 in univariate analysis were included in multivariate regression analysis for correction to explore whether baseline NIHSS, Pre-ASPECTS, and Post-ASPECTS can predict 90-day prognosis, 90-day death, and HT. Furthermore, receiver operating characteristic (ROC) curve analysis was performed to determine the best predictive value. The R 4.0.4 software was used to draw ROC plots. The MedCalc 20.106 software was used to perform the DeLong test analysis. A *P*-value of < 0.05 was considered to indicate a statistically significant difference.

## Results

### General information

**Figure 1:**
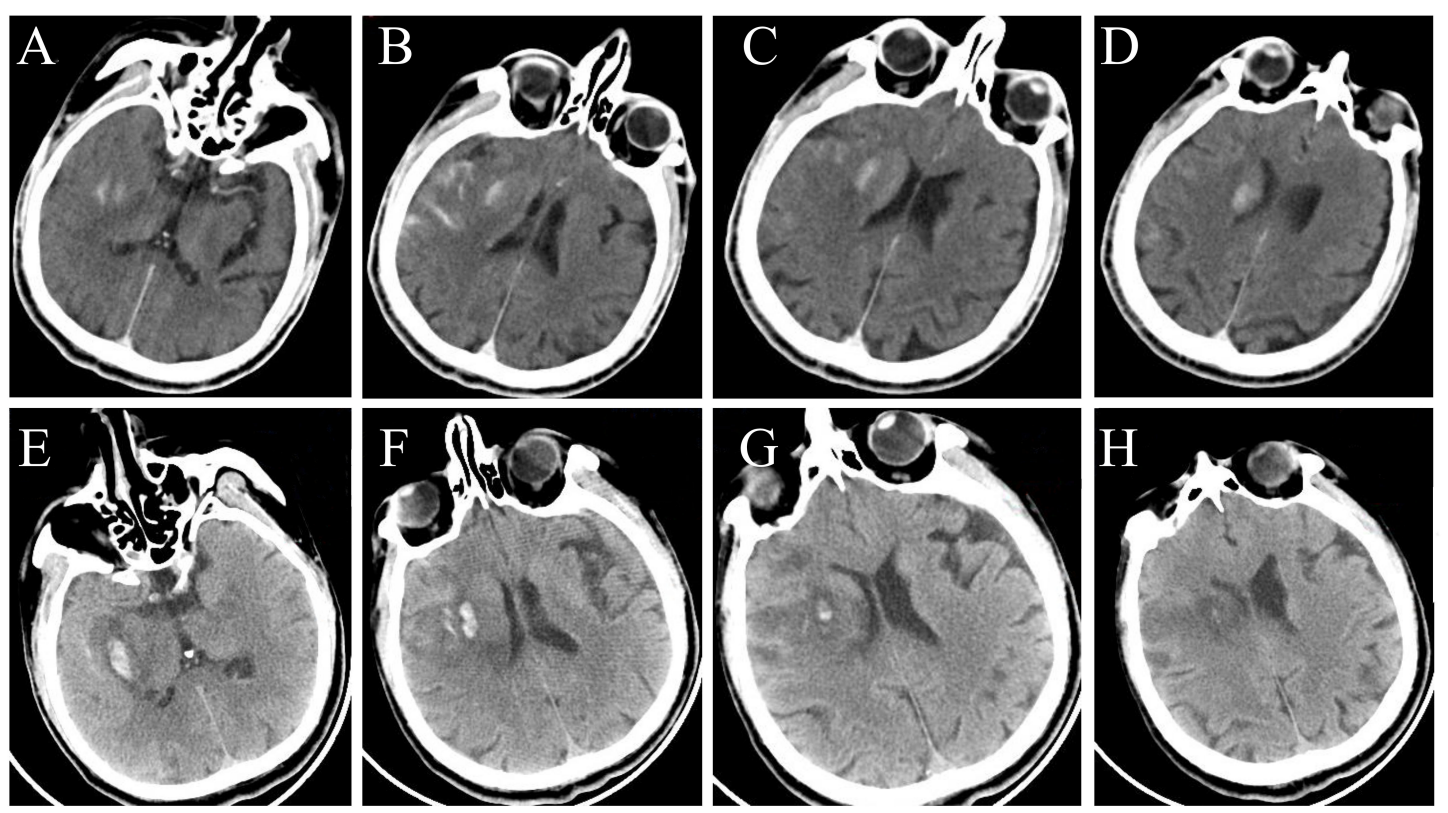
Representative images of parenchymal hyperdensity immediately after thrombectomy on NCCT **(A–D)** for Post-ASPECTS assessment; and NCCT images **(E–H)** at 5 days after thrombectomy showing HT. NCCT, non-contrast computed tomography; Post-ASPECTS, post-thrombectomy ASPECTS; HT, hemorrhagic transformation.

A total of 231 patients who met all of the inclusion and exclusion criteria underwent thrombectomy for acute anterior-circulation ischemic stroke within 10 h. The study participants comprised 132 males (57.1%) and 99 females (42.9%), with a median age of 69 years (IQR, 61–75 years). Among the 231 patients, 48 patients (20.8%) received intravenous thrombolysis before thrombectomy. The OTP time was 0.9–9.6 h. A total of 204 patients (88.3%) underwent thrombectomy within 6 h, and 27 patients (11.7%) underwent thrombectomy between 6.0 and 9.6 h. The incidence of post-thrombectomy parenchymal hyperdensity was 79.2%. A total of 22 patients were without CT to judge HT 7 days after thrombectomy, so 209 patients were included in the HT statistics. The good prognosis rate, mortality rate, and HT rate were 57.1, 9.5, and 38.3% (80/209), respectively. Representative images of parenchymal hyperdensity after thrombectomy to evaluate Post-ASPECTS showed a baseline NIHSS score of 17, an OTP time of 9.6 h, Pre-ASPECTS of 9, and Post-ASPECTS of 5; the mRS score at 90 days was 3 (Figure 1).

### Factors influencing patient outcome analysis

There were no significant differences in sex, hypertension, intravenous thrombolysis, OTP time, or OTR time between the good prognosis group (132, 57.1%) and the poor prognosis group (99, 42.9%). Compared to the parameters of the good prognosis group, patients in the poor prognosis group were older (Z = −2.586, *P* = 0.010) and had higher baseline NIHSS scores (Z = −6.473, *P* < 0.001) and ICA (37.4 vs. 23.5%) and HT (55.3 vs. 26.6%, *P* < 0.001) rates. The patients in the poor prognosis also had a higher proportion of diabetes (21.2 vs. 10.6%, *P* = 0.026) and atrial fibrillation (58.6 vs. 39.4%, *P* = 0.004), a longer hospital stay (Z = −4.149, *P* < 0.001), as well as lower Pre-ASPECTS (Z = −3.022, *P* = 0.003) and Post-ASPECTS (Z = −7.082, *P* < 0.001) (Table 1).

**Table 1:** Comparison of the good prognosis group with the poor prognosis group at the 90-day follow-up.

	**90-day mRS**		**Z/χ2 Value**	***P-*Value**
	**mRS 0–2 (*n* = 132)**	**mRS 3–6 (*n* = 99)**		
Age, median (IQR), y	68 (59–74)	71 (64–78)	−2.586	0.010
Men sex, *n* (%)	81 (61.4)	51 (51.5)	2.241	0.134
Smoking, *n* (%)	41 (31.1)	11 (11.1)	12.907	<0.001
Hypertension, *n* (%)	77 (58.3)	59 (59.6)	0.037	0.847
Diabetes, *n* (%)	14 (10.6)	21 (21.2)	4.950	0.026
Atrial fibrillation, n (%)	52 (39.4)	58 (58.6)	8.354	0.004
Baseline NIHSS, median (IQR)	12 (11–15)	16 (13–20)	−6.473	<0.001
Pre-ASPECTS, median (IQR)	10 (8–10)	9 (8–10)	−3.022	0.003
Intravenous thrombolysis, n (%)	29 (22.0)	19 (19.2)	0.265	0.607
OTP time, median (IQR), min	280 (220–329)	265 (210–334)	−0.082	0.935
OTR time, median (IQR), min	344 (276–394)	345 (285–415)	−1.048	0.295
Post-ASPECTS, median (IQR)	8 (7–10)	6 (4–7)	−7.082	<0.001
Length of hospital stay, median (IQR)	9 (7–12)	12 (8–21)	−4.149	<0.001
**Occlusion artery**, ***n*** **(%)**			6.605	0.037
ICA	31 (23.5) a	37 (37.4) b		
M1	87 (65.9) a	49 (49.5) b		
ICA + M1	14 (10.6) a	13 (13.1) a		
**HT**, ***n*** **(%)**	33/124 (26.6)	47/85 (55.3)	17.559	<0.001
HI1	11 (8.9) a	9 (10.6) a		
HI2	17 (13.7) a	16 (18.8) a		
PH1	4 (3.2) a	5 (5.9) a		
PH2	1 (0.8) a	17 (20.0) b		

*Different letters (a and b) represent statistical differences between groups. Pre-ASPECTS, Pre-thrombectomy ASPECTS; OTP, onset to groin puncture; OTR, onset to recanalization; Post-ASPECTS, post-thrombectomy ASPECTS; ICA, internal carotid artery; M1, M1 segment of the middle cerebral artery; HT, hemorrhagic transformation*.

The death group (22, 9.5%) had a higher baseline NIHSS score (Z = −4.001, *P* < 0.001), diabetes rate (36.4 vs. 12.9%, *P* = 0.009), and a longer hospital stay (Z = −2.802, *P* = 0.005) than the non-death group (209, 90.5%). The Pre-ASPECTS (Z = −4.086, *P* < 0.001) and Post-ASPECTS (Z = −3.875, *P* < 0.001) were lower in the death group (Supplementary Table S1).

In the HT group, 20 patients (25.0%) received intravenous thrombolysis, although there was no significant difference in the thrombolysis rate between the HT and non-HT groups. The HT group (80, 38.3%) had a higher baseline NIHSS score (Z = −2.725, *P* = 0.006), longer hospital stay (Z = −3.930, *P* < 0.001) and OTR time (Z = −2.733, *P* = 0.006), and lower Pre-ASPECTS (Z = −2.531, *P* = 0.011) and Post-ASPECTS (Z = −7.660, *P* < 0.001), compared to the non-HT group (129, 61.7%) (Supplementary Table S2).

### Correlation and regression analysis of 90-day poor prognosis, 90-day death, and HT

The results of linear correlation analysis showed that there were correlations between baseline NIHSS score, Pre-ASPECTS, Post-ASPECTS, and length of hospital stay with 90-day mRS scores, in which Post-ASPECTS had the highest correlation coefficient (−0.479, *P* < 0.01, Table 2).

**Table 2:** Correlation analysis of 90-day prognosis.

**90-day mRS**	**Correlation coefficient**
Baseline NIHSS	0.441^**^
Pre-ASPECTS	−0.238^**^
Post-ASPECTS	−0.479^**^
OTP time	0.013
OTR time	0.091
Length of hospital stay	0.262^**^

** *P < 0.01. Pre-ASPECTS, pre-thrombectomy ASPECTS; Post-ASPECTS, post-thrombectomy ASPECTS; OTP, onset to groin puncture; OTR, onset to recanalization*.

In the first multivariable logistic regression analysis model, both baseline NIHSS score (aOR, 1.240, 95% CI, 1.127–1.366; *P* < 0.001) and the Pre-ASPECTS (aOR, 0.608, 95% CI, 0.438–0.844; *P* = 0.003) affected poor prognosis; both baseline NIHSS score (aOR, 1.171, 95% CI, 1.068–1.283; *P* = 0.001) and the Pre-ASPECTS (aOR, 0.394, 95% CI, 0.245–0.635; *P* < 0.001) affected death, while only the adjusted Pre-ASPECTS (aOR, 0.641, 95% CI, 0.488–0.840; *P* = 0.001) affected HT. In the second multivariable logistic regression analysis model, the baseline NIHSS score (aOR, 1.222, 95% CI, 1.107–1.348; *P* < 0.001), the Pre-ASPECTS (aOR, 0.678, 95% CI, 0.477–0.962; *P* = 0.030), and the Post-ASPECTS (aOR, 0.666, 95% CI, 0.525–0.845; *P* = 0.001) affected poor prognosis; the baseline NIHSS score (aOR, 1.147, 95% CI, 1.040–1.265; *P* = 0.006), the Pre-ASPECTS (aOR, 0.453, 95% CI, 0.276–0.746; *P* = 0.002), and the Post-ASPECTS (aOR, 0.693, 95% CI, 0.529–0.908; *P* = 0.008) affected death, while only the adjusted Post-ASPECTS (aOR, 0.607, 95% CI, 0.496–0.743; *P* < 0.001) affected HT (Table 3).

**Table 3:** Multivariable logistic regression analysis of 90-day poor prognosis, 90-day death, and HT.

	**Logistic regression**					
	**Crude OR** ** (95% CI)**	**Crude *P-*value**	**Model 1**	**Model 1**	**Model 2**	**Model 2**
			**Adjusted OR** ** (95% CI)**	**Adjusted *P-*value**	**Adjusted OR ** **(95% CI)**	**Adjusted *P-*value**
**90-day mRS 3–6**						
Baseline NIHSS	1.238 (1.146–1.338)	<0.001	1.240 (1.127–1.366)^a^	<0.001	1.222 (1.107–1.348)^c^	<0.001
Pre-ASPECTS	0.672 (0.531–0.850)	<0.001	0.608 (0.438–0.844)^b^	0.003	0.678 (0.477–0.962)^d^	0.030
Post-ASPECTS	0.589 (0.498–0.696)	<0.001	Not included	-	0.666 (0.525–0.845)^e^	0.001
**90-day death**						
Baseline NIHSS	1.167 (1.087–1.253)	<0.001	1.171 (1.068–1.283)^f^	0.001	1.147 (1.040–1.265)^h^	0.006
Pre-ASPECTS	0.464 (0.319–0.675)	<0.001	0.394 (0.245–0.635)^g^	<0.001	0.453 (0.276–0.746)^i^	0.002
Post-ASPECTS	0.658 (0.545–0.794)	<0.001	Not included	-	0.693 (0.529–0.908)^j^	0.008
**HT**						
Baseline NIHSS	1.084 (1.024–1.147)	0.005	1.062 (0.999–1.130)^k^	0.054	1.019 (0.950–1.094)^m^	0.598
Pre-ASPECTS	0.694 (0.543–0.889)	0.004	0.641 (0.488–0.840)^l^	0.001	0.785 (0.579–1.064)^n^	0.119
Post-ASPECTS	0.551 (0.458–0.662)	<0.001	Not included	-	0.607 (0.496–0.743)°	<0.001

a *Adjusted for age, Pre-ASPECTS, smoking, diabetes, atrial fibrillation, length of hospital stay, occlusion artery, and HT;*

b *adjusted for age, baseline NIHSS score, smoking, diabetes, atrial fibrillation, length of hospital stay, occlusion artery, and HT;*

c *adjusted for age, Pre-ASPECTS, Post-ASPECTS, smoking, diabetes, atrial fibrillation, length of hospital stay, occlusion artery, and HT;*

d *adjusted for age, baseline NIHSS score, Post-ASPECTS, smoking, diabetes, atrial fibrillation, length of hospital stay, occlusion artery, and HT;*

e *adjusted for age, baseline NIHSS score, Pre-ASPECTS, smoking, diabetes, atrial fibrillation, length of hospital stay, occlusion artery, and HT;*

f *adjusted for age, Pre-ASPECTS, diabetes, length of hospital stay, and HT;*

g *adjusted for age, baseline NIHSS score, diabetes, length of hospital stay, and HT;*

h *adjusted for age, Pre-ASPECTS, Post-ASPECTS, diabetes, length of hospital stay, and HT;*

i *adjusted for age, baseline NIHSS score, Post-ASPECTS, diabetes, length of hospital stay, and HT;*

j *adjusted for age, baseline NIHSS score, Pre-ASPECTS, diabetes, length of hospital stay, and HT;*

k *adjusted for age, Pre-ASPECTS, OTR, and length of hospital stay;*

l *adjusted for age, baseline NIHSS score, OTR, and length of hospital stay;*

m *adjusted for age, Pre-ASPECTS, Post-ASPECTS, OTR, and length of hospital stay;*

n *adjusted for age, baseline NIHSS score, Post-ASPECTS, OTR, and length of hospital stay; °adjusted for age, baseline NIHSS score, Pre-ASPECTS, OTR, and length of hospital stay. HT, hemorrhagic transformation; Post-ASPECTS, post-thrombectomy ASPECTS*.

### ROC curve analysis of the best predictive value

**Figure 2:**
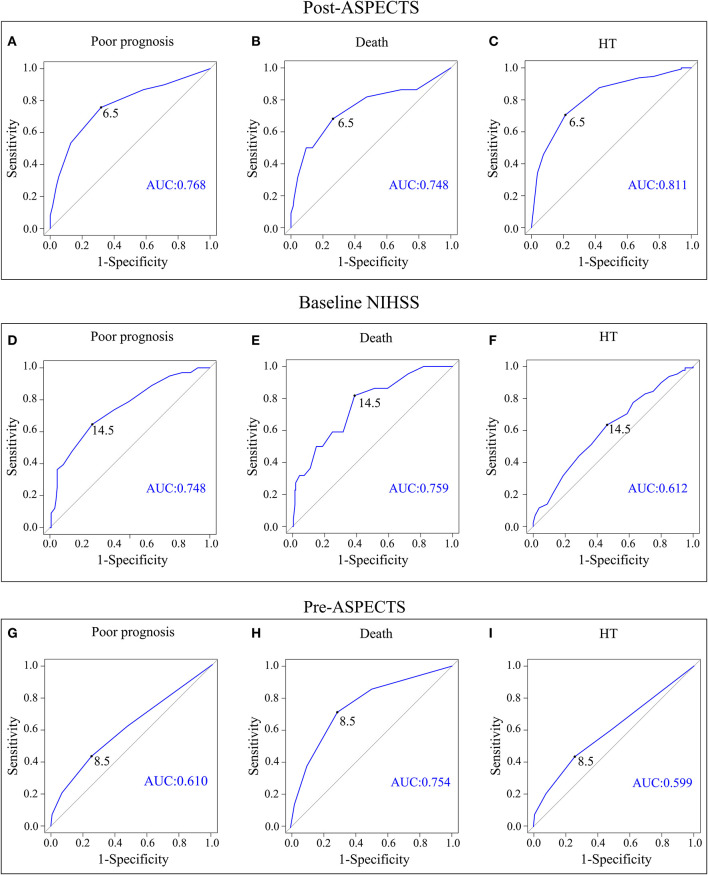
ROC curve analysis plots of the Post-ASPECTS with 90-day poor prognosis **(A)**, 90-day death **(B)**, and HT **(C)**. The baseline NIHSS score with 90-day poor prognosis **(D)**, 90-day death **(E)**, and HT **(F)**. The Pre-ASPECTS with 90-day poor prognosis **(G)**, 90-day death **(H)**, and HT **(I)**. ROC, receiver operating characteristic; Pre-ASPECTS, pre-thrombectomy ASPECTS; Post-ASPECTS, post-thrombectomy ASPECTS; HT, hemorrhagic transformation.

Receiver operating characteristic curve analysis showed that the optimal cutoff value of the baseline NIHSS score (> 14) could predict poor outcome (AUC, 0.748; *P* < 0.001), death (AUC, 0.759; *P* < 0.001), and HT (AUC, 0.612; *P* = 0.007); the optimal cutoff value of the Pre-ASPECTS (< 9) could predict poor outcome (AUC, 0.610; *P* = 0.005), death (AUC, 0.754; *P* < 0.001), and HT (AUC, 0.599; *P* = 0.018). Moreover, Post-ASPECTS < 7 could predict poor prognosis (AUC, 0.768; *P* < 0.001), death (AUC, 0.748; *P* < 0.001), and HT (AUC, 0.811; *P* < 0.001) (Table 4, Figure 2). The results of the Delong test showed that there was no statistical difference between baseline NIHSS and Post-ASPECTS in predicting poor prognosis (*P* > 0.05) and death (*P* > 0.05), but a statistical difference in predicting HT (*P* < 0.001). There was no significant difference between the Pre-ASPECTS and Post-ASPECTS in predicting death (*P* > 0.05), but there was a significant difference in predicting poor prognosis and HT (*P* < 0.05), which indicates that Post-ASPECTS has better predictive power (Figure 2). The baseline NIHSS score (cutoff value > 14) could predict poor prognosis, death, and HT, with sensitivities of 64.6, 81.8, and 53.8%, specificities of 73.5, 61.2, and 63.6%, and positive predictive values of 64.6, 18.2, and 47.8%, respectively. The Post-ASPECTS (cutoff value < 7) could predict poor prognosis, death, and HT, with sensitivities of 53.5, 68.2, and 57.5%, specificities of 87.1, 73.7, and 87.6%, and positive predictive values of 75.7, 21.4, and 74.2%, respectively. The Post-ASPECTS for predicting poor prognosis and death had higher specificity with lower sensitivity than the baseline NIHSS score. Additionally, Post-ASPECTS could also predict HT with higher predictive power (AUC = 0.811) and specificity (Table 4).

**Table 4:** ROC curve for 90-day prognosis, death, and HT.

.	**AUC**	**Best predictive** ** value**	**Sensitivity** ** (%)**	**Specificity** ** (%)**	**Positive predictive** ** value**	**Negative predictive** ** value**	**95% CI**	***P* value**
**Baseline NIHSS**								
Poor prognosis	0.748	>14	64.6	73.5	64.6	73.5	0.685–0.812	<0.001
Death	0.759	>14	81.8	61.2	18.2	97.0	0.657–0.860	<0.001
HT	0.612	>14	53.8	63.6	47.8	68.9	0.534–0.690	0.007
**Pre-ASPECTS**								
Poor prognosis	0.610	<9	43.3	74.8	56.0	64.1	0.535–0.685	0.005
Death	0.754	<9	71.4	71.0	20.0	96.1	0.643–0.865	<0.001
HT	0.599	<9	43.6	74.2	50.7	68.3	0.517–0.680	0.018
**Post-ASPECTS**								
Poor prognosis	0.768	<7	53.5	87.1	75.7	71.4	0.705–0.832	<0.001
Death	0.748	<7	68.2	73.7	21.4	95.7	0.620–0.875	<0.001
HT	0.811	<7	57.5	87.6	74.2	76.9	0.751–0.870	<0.001

*ROC, receiver operating characteristic; HT, hemorrhagic transformation; Post-ASPECTS, post-thrombectomy ASPECTS*.

### Different prognosis of patients grouped by baseline NIHSS score and Post-ASPECTS

Compared with the baseline NIHSS score > 14 group, the baseline NIHSS score ≤ 14 group had a lower proportion of poor prognosis, mortality, and HT. Compared to the Post-ASPECTS < 7 group, the Post-ASPECTS ≥ 7 group had a lower proportion of poor prognosis, mortality, and HT. Thereafter, a combined Post-ASPECTS with baseline NIHSS score for prognosis prediction was used, which showed that the percentages of poor prognosis, mortality, and HT were 87.5, 30.0, and 80.6%, respectively, in the NIHSS > 14 + Post-ASPECTS < 7 group. In contrast, the percentages of poor prognosis, mortality, and HT were 16.7, 1.0, and 21.5%, respectively, in the NIHSS ≤ 14 + Post-ASPECTS ≥ 7 group (Table 5).

**Table 5:** Baseline NIHSS score and Post-ASPECTS predict patient prognosis.

	**Post-ASPECTS** **(*****n*** ***=*** **231)**		**Baseline NIHSS** **(*****n*** ***=*** **231)**		**Baseline NIHSS** + **Post-ASPECTS** **(*****n*** **=** **231)**		
	** <7** ** (*n =* 70)**	**≥7** ** (*n =* 161)**	** ≤ 14** ** (*n =* 132)**	**>14** ** (*n =* 99)**	** ≤ 14 + ≥7** ** (*n =* 102)**	**>14 + <7** ** (*n =* 40)**	**Others** ** (*n =* 89)**
Poor prognosis, n (%)	53 (75.7)	46 (28.6)^***^	35 (26.5)	64 (64.6)^***^	17 (16.7) a	35 (87.5) b	47 (52.8) c^***^
Death, n (%)	15 (21.4)	7 (4.3)^***^	4 (3.0)	18 (18.2)^***^	1 (1.0) a	12 (30.0) b	9 (10.1) c^***^
HT, n (%)	46/62 (74.2)	34/147 (23.1)^***^	37/119 (31.1)	43/90 (47.8)^*^	20/93 (21.5) a	29/36 (80.6) b	31/80 (38.8) c^***^

* *P < 0.05;*

*** *P < 0.001. Different letters (a, b, and c) represent statistical differences between groups*.

*Post-ASPECTS, post-thrombectomy ASPECTS; HT, hemorrhagic transformation*.

## Discussion

This study aimed to investigate the association of Post-ASPECTS based on brain parenchymal hyperdensity within 1 h after thrombectomy (refer to ASPECTS scoring method) with prognosis, death, and HT. The main findings were that Post-ASPECTS could predict 90-day prognosis, 90-day death, and HT with high specificity and positive predictive values in patients with ACLVO undergoing thrombectomy.

Age, collateral circulation, OTP time, and OTR time can all affect outcomes after thrombectomy (11, 22). Preoperative stroke severity is another indicator of clinical outcomes, and severe stroke (NIHSS > 14) is an independent predictor of a poor 90-day prognosis (23, 24). In this study, we also found that the baseline NIHSS score could predict a 90-day poor prognosis, with > 14 found to be the optimal cutoff value; in addition, baseline NIHSS could predict 90-day death and HT. The adjusted Pre-ASPECTS could predict 90-day poor prognosis and 90-day death. The Delong test showed that the Post-ASPECTS was better than the Pre-ASPECTS in predicting the 90-day poor prognosis (*P* < 0.05), and the Post-ASPECTS was better than baseline NIHSS and Pre-ASPECTS in predicting HT (*P* < 0.05).

With the development of thrombectomy technology, the recanalization rate has approached 95% (8, 9). However, the percentage of good prognosis in clinical trials is 71% at best, whereas approximately half of the population have futile recanalization (8, 10). In our study, the good prognosis rate was 57.1%, which is consistent with that of previous findings (2, 3, 10). To date, there are no suitable preoperative indicators that can accurately predict which patients will suffer futile recanalization; therefore, an early postoperative indicator of prognosis helps to guide clinical treatment. The prognosis predictive index after thrombectomy is the current focus of clinicians, and there are some related studies.

It has been reported that postoperative indicators such as RNI, cerebral tissue reperfusion examinations, and follow-up infarct volumes could predict prognosis (4, 12, 14, 25, 26), whereas the presence of anesthesia, sedation, hyperthermia, and a “stunned brain” affects the assessment of NIHSS scores during the early postoperative period (27). Thus, RNI is generally assessed at 24 h after thrombectomy, and the follow-up of infarct volumes is performed later (generally between 24 h and 1 week) (14, 25, 26). The evaluation of perfusion status by CTP or MRI within 24 h after thrombolysis or thrombectomy can also predict prognosis, while recanalization cannot (5, 28). However, perfusion status examinations exhibit considerable technical difficulties, making them difficult to apply to some patients and hospitals, especially in clinical practice.

Non-contrast computed tomography is a routine examination after thrombectomy, and brain parenchymal hyperdensity is a common imaging manifestation. The incidence of brain parenchymal hyperdensity after thrombectomy has been reported to vary from 60.8 to 84.2%, which is related to whether the artery is successfully recanalized as well as the time of CT examinations (17, 19, 29, 30). In this study, we restricted our research objects to successful recanalization, and NCCT was completed immediately (within 1 h) after thrombectomy. As such, the incidence of post-thrombectomy parenchymal hyperdensity was 79.2%.

Post-thrombectomy parenchymal hyperdensity contains contrast staining and hemorrhage, and most previous studies focus on contrast staining mainly. Some studies have found that contrast staining is associated with HT (29) and poor prognosis (15, 31). However, most studies have suggested that contrast staining cannot predict prognosis (16, 17, 19). Both contrast staining and hemorrhage are thought to be associated with the breakdown of the blood-brain barrier (BBB) (16, 31, 32). In Desilles' study, the brain parenchymal hyperdensity on NCCT immediately after thrombectomy was considered as the disruption of BBB (33). It has been reported that disruption of the BBB is associated with the progression of infarct volumes and neurobehavioral severities (34). For these reasons, we did not distinguish contrast staining from hemorrhage and only excluded obvious occupying lesions for highly predictive bleeding. In addition, previous studies on brain parenchymal hyperdensity generally conducted qualitative analysis (32). Instead, semiquantitative Post-ASPECTS was used in our study according to the ASPECTS scoring method (20). Our results show that Post-ASPECTS is better than two preoperative factors (baseline NIHSS and Pre-ASPECTS) in predicting HT, perhaps because Post-ASPECTS can reflect BBB disruption.

Interestingly, we found that Post-ASPECTS not only predicted HT, but it was also an independent predictor of prognosis and death. The specificity for predicting HT reached 87.6%, which was essentially comparable to that of dual-energy CT (84.4–100.0%) (35, 36). Additionally, its specificities for predicting poor prognosis and death were 87.1 and 73.7%, respectively. It has been reported that the highest specificity of final infarct volume (>90 cm^3^) for poor outcome was approximately 90%, which was similar to our results (37). The Post-ASPECTS < 7 group had a high rate of HT of 74.2%, a high poor prognosis rate of 75.7%, and a mortality rate of 21.4%. It has been reported that the poor outcome rate was 63.0%, and the mortality rate was 18.0% in the RNI group (12). Moreover, the poor outcome rate was approximately 68.8% in the no reperfusion group when perfusion examination was used to predict prognosis (38). Desilles' study showed that BBB disruption based on brain parenchymal hyperdensity was related to prognosis and HT, while patients with BBB disruption had a poor prognosis rate of 60.2% and hemorrhagic complications of 42.2%, which were lower than those observed in our study. Compared to these data, we find that semiquantitative Post-ASPECTS based on brain parenchymal hyperdensity have a relatively higher positive predictive value.

In conclusion, we determined that the semiquantitative Post-ASPECTS based on brain parenchymal hyperdensity in immediate postoperative NCCT was effective in predicting 90-day poor prognosis, death, and HT in patients with AIS with ACLVO, with an optimal cutoff value of 7. Post-ASPECTS is better than baseline NIHSS in predicting HT and better than Pre-ASPECTS in predicting 90-day poor prognosis and HT. The Post-ASPECTS is easy to perform without patient cooperation and additional checking instruments.

Our study does have some shortcomings considering its retrospective design and the small number of included cases, which may lead to selective bias and needs to be confirmed by more studies in the near future. Nevertheless, our results provide a valuable evaluation index for ultra-early prediction of prognosis with high specificity and high positive predictive values in patients with AIS with ACLVO after successful recanalization.

## Data availability statement

The original contributions presented in the study are included in the article/Supplementary material, further inquiries can be directed to the corresponding author.

## Ethics statement

The studies involving human participants were reviewed and approved by the Institutional Review Board at First Affiliated Hospital of Anhui Medical University. Written informed consent for participation was not required for this study in accordance with the national legislation and the institutional requirements.

## Author contributions

LLC drafted the manuscript. LLC and YCJ participated in data collection. CZ participated in the data analysis. LLC, ZOX, YCJ, XJH, and JYW evaluated Pre-ASPECTS and Post-ASPECTS. ZQX, XJH, WMY, ZMZ, SPW, and JYW performed the thrombectomies. KW, BYL, and JYW participated in study design and manuscript revision. All of the authors reviewed and approved the final manuscript.

## Funding

This study was supported by a grant from the National Natural Science Foundation of China (81870918), the National Key Research and Development Program of China (2016YFC1300604), and Zhejiang province's basic public welfare projects (LGF20H090010).

## Conflict of interest

The authors declare that the research was conducted in the absence of any commercial or financial relationships that could be construed as a potential conflict of interest.

## Publisher's note

All claims expressed in this article are solely those of the authors and do not necessarily represent those of their affiliated organizations, or those of the publisher, the editors and the reviewers. Any product that may be evaluated in this article, or claim that may be made by its manufacturer, is not guaranteed or endorsed by the publisher.

## Abbreviations

ACLVO: anterior-circulation large vessel occlusion
AIS: acute ischemic stroke
BBB: blood-brain barrier
HT: hemorrhagic transformation
ICA: internal carotid artery
M1: M1 segment of middle cerebral artery
NCCT: non-contrast computed tomography
OTP: onset to groin puncture
OTR: onset to recanalization
Post-ASPECTS: post-thrombectomy ASPECTS
Pre-ASPECTS: pre-thrombectomy ASPECTS
RNI: rapid neurological improvement
ROC: receiver operating characteristic.
