# Supplementary material for: Post-ASPECTS based on hyperdensity in NCCT immediately after thrombectomy is an ultra-early predictor of hemorrhagic transformation and prognosis
Source: Front Neurol. 2022 Aug 10;13:887277. doi: 10.3389/fneur.2022.887277 (PMC9399794; doi:10.3389/fneur.2022.887277)
Supplement: Supplementary file 1 [file Data_Sheet_1.DOCX]

**SUPPLEMENTAL MATERIAL**

**Table 1. Comparison of the non-death group with the death group at the 90-Day Follow-Up**

|  | mRS 0–5 | | mRS 6 | **Z/χ2 Value** | ***P* Value** |
| --- | --- | --- | --- | --- | --- |
|  | n = 209 | n = 22 | |  |  |
| Age, median (IQR), y | 69 (61–75) | 71 (61–79) | | –0.550 | 0.582 |
| Men sex, n (%) | 122 (58.4%) | 10 (45.5%) | | 1.356 | 0.244 |
| Smoking, n (%) | 48 (23.0%) | 4 (18.2%) | | 0.261 | 0.609 |
| Hypertension, n (%) | 121 (57.9%) | 15 (68.2%) | | 0.870 | 0.351 |
| Diabetes, n (%) | 27 (12.9%) | 8 (36.4%) | | 6.785 | 0.009 |
| Atrial fibrillation, n (%) | 96 (45.9%) | 14 (63.6%) | | 2.501 | 0.114 |
| Baseline NIHSS, median (IQR) | 14 (11–17) | 18 (15–25) | | –4.001 | <0.001 |
| Pre-ASPECTS, median (IQR) | 9 (8–10) | 8 (7–9) | | –4.086 | <0.001 |
| Intravenous thrombolysis, n (%) | 43 (20.6%) | 5 (22.7%) | | 0.056 | 0.813 |
| OTP time, median (IQR), min | 280 (220–330) | 245 (190–329) | | –0.903 | 0.367 |
| OTR time, median (IQR), min | 345 (281–405) | 345 (275–403) | | –0.614 | 0.539 |
| Post-ASPECTS, Median (IQR) | 8 (6–9) | 5 (3–7) | | –3.875 | <0.001 |
| Length of hospital stay, median (IQR) | 10 (8–15) | 6 (3–14) | | –2.802 | 0.005 |
| **Occlusion artery, n (%)** |  |  | | 0.187 | 0.911 |
| ICA | 61 (29.2%) | 7 (31.8%) | |  |  |
| M1 | 123 (58.9%) | 13 (59.1%) | |  |  |
| ICA + M1 | 25 (12.0%) | 2 (9.1%) | |  |  |
| **HT, n (%)** | 69/188 (36.7%) | 11/21 (52.4%) | | 1.965 | 0.161 |
| HI1 | 18 (9.6%) | 2 (9.5%) | |  |  |
| HI2 | 29 (15.4%) | 4 (19.0%) | |  |  |
| PH1 | 8 (4.3%) | 1 (4.8%) | |  |  |
| PH2 | 14 (7.4%) | 4 (19.0%) | |  |  |

Pre-ASPECTS: Pre-thrombectomy ASPECTS, OTP: Onset to groin puncture, OTR: Onset to recanalization, Post-ASPECTS: Post-thrombectomy ASPECTS, ICA: Internal carotid artery, M1: M1 segment of middle cerebral artery, HT: Hemorrhagic transformation.

**Table 2. Comparison of the HT group with the non-HT group**

|  | **HT** | | **Z/χ2 Value** | ***P* Value** |
| --- | --- | --- | --- | --- |
|  | Non-HT (n = 129) | HT (n = 80) |  |  |
| Age, median (IQR), y | 68 (61–76) | 70 (61–75) | –0.379 | 0.705 |
| Men sex, n (%) | 78 (60.5%) | 42 (52.5%) | 1.281 | 0.258 |
| Smoking, n (%) | 33 (25.6%) | 16 (20.0%) | 0.857 | 0.355 |
| Hypertension, n (%) | 74 (57.4%) | 44 (55.0%) | 0.112 | 0.738 |
| Diabetes, n (%) | 18 (14.0%) | 13 (16.3%) | 0.206 | 0.650 |
| Atrial fibrillation, n (%) | 59 (45.7%) | 40 (50.0%) | 0.360 | 0.548 |
| Baseline NIHSS, median (IQR) | 13 (11–16) | 15 (12–19) | –2.725 | 0.006 |
| Pre-ASPECTS, median (IQR) | 10 (8–10) | 9 (8–10) | –2.531 | 0.011 |
| Intravenous thrombolysis, n (%) | 24 (18.6%) | 20 (25.0%) | 1.215 | 0.270 |
| OTP time, median (IQR), min | 270 (195–320) | 298 (233–339) | –1.855 | 0.064 |
| OTR time, median (IQR), min | 330 (261–380) | 353 (290–429) | –2.733 | 0.006 |
| Post-ASPECTS, median (IQR) | 8 (7–10) | 6 (5–7) | –7.660 | <0.001 |
| Length of hospital stay, median (IQR) | 9 (7–13) | 12 (9–19) | –3.930 | <0.001 |
| Occlusion artery, n (%) |  |  | 5.701 | 0.058 |
| ICA | 36 (27.9%) | 25 (31.3%) |  |  |
| M1 | 83 (64.3%) | 41 (51.3%) |  |  |
| ICA + M1 | 10 (7.8%) | 14 (17.5%) |  |  |

HT: Hemorrhagic transformation, Pre-ASPECTS: Pre-thrombectomy ASPECTS, OTP: Onset to groin puncture, OTR: Onset to recanalization, Post-ASPECTS: Post-thrombectomy ASPECTS, ICA: Internal carotid artery, M1: M1 segment of middle cerebral artery.

**Comparison of Post-ASPECTS and Final Infarct Volume in Predicting 90-day Prognosis**

It is very meaningful to compare the differences between Post-ASPECTS score and final infarct volume in predicting patients' 90-day prognosis, as suggested by the reviewer. Unfortunately, it was not considered in the initial design of the study. To illustrate this point, one single-center (the First Affiliated Hospital of Anhui Medical University) results were supplemented here. 67 patients who had CT or MR DWI examination 2 days after onset to evaluate the infarct volume were selected. The infarct volume was also scored according to ASPECTS methods (IV-ASPECTS). ROC curve analysis showed that both the Post-ASPECTS score and IV-ASPECTS score could predict the 90-day poor prognosis, but there was no significant difference in the predictive value (*P* = 0.11) (Figure 1).

Although there is no significant difference between the Post-ASPECTS score and final infarct volume in predicting 90-day prognosis, the Post-ASPECTS score can predict poor prognosis immediately after the operation, which helps to formulate treatment plans as soon as possible. For example, some patients can undergo bone flap decompression surgery in advance to improve their prognosis. In addition, it also helps doctors and patients' families predict the prognosis of patients in advance and give up some ineffective treatment in the early stage.


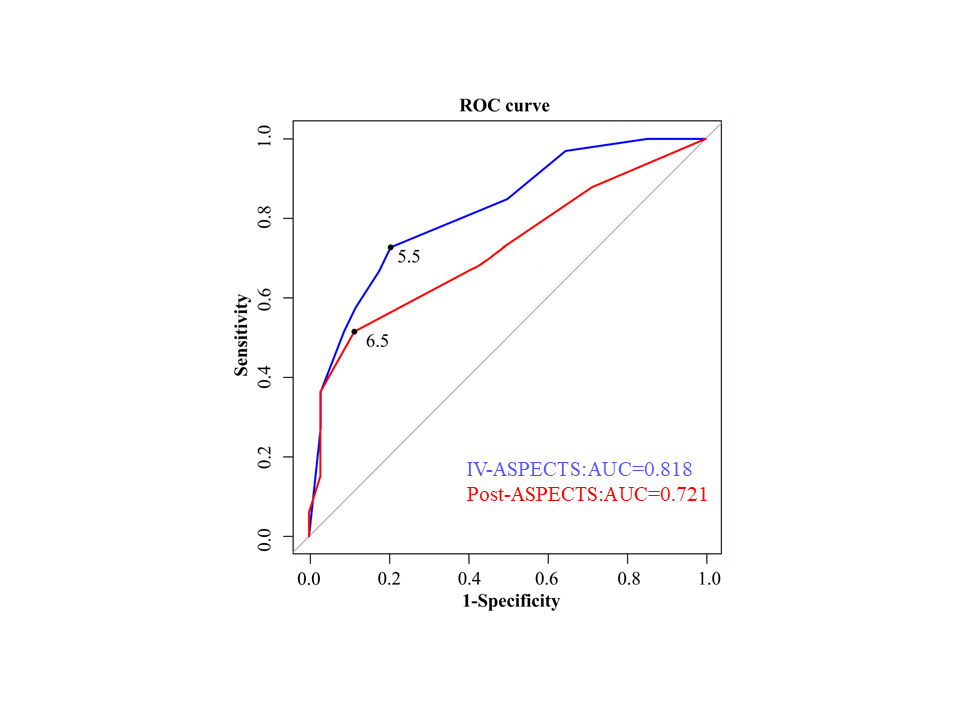


**Figure 1.** ROC curve analysis plots of Post-ASPECTS score and IV-ASPECTS score in predicting 90-day prognosis, respectively. Post-ASPECTS: Post-thrombectomy ASPECTS, IV-ASPECTS: infarct volume ASPECTS
